# Impaired holistic processing of left-right composite faces in congenital prosopagnosia

**DOI:** 10.3389/fnhum.2014.00750

**Published:** 2014-09-29

**Authors:** Tina T. Liu, Marlene Behrmann

**Affiliations:** ^1^Department of Psychology, Carnegie Mellon UniversityPittsburgh, PA, USA; ^2^Center for the Neural Basis of Cognition, Carnegie Mellon UniversityPittsburgh, PA, USA

**Keywords:** congenital prosopagnosia, holistic processing, composite face effect, chimeric face, face lateralization

## Abstract

Congenital prosopagnosia (CP) refers to a lifelong impairment in face processing despite normal visual and intellectual skills. Many studies have suggested that the key underlying deficit in CP is one of a failure to engage holistic processing. Moreover, there has been some suggestion that, in normal observers, there may be greater involvement of the right than left hemisphere in holistic processing. To examine the proposed deficit in holistic processing and its potential hemispheric atypicality in CP, we compared the performance of 8 CP individuals with both matched controls and a large group of non-matched controls on a novel, vertical composite task. In this task, participants judged whether a cued half of a face (either left or right half) was the same or different at study and test, and the two face halves could be either aligned or misaligned. The standard index of holistic processing is one in which the unattended face half influences performance on the cued half and this influence is greater in the aligned than in the misaligned condition. Relative to controls, the CP participants, both at a group and at an individual level, did not show holistic processing in the vertical composite task. There was also no difference in performance as a function of hemifield of the cued face half in the CP individuals, and this was true in the control participants, as well. The findings clearly confirm the deficit in holistic processing in CP and reveal the useful application of this novel experimental paradigm to this population and potentially to others as well.

## Introduction

Congenital prosopagnosia (CP) refers to the apparently lifelong impairment in face recognition despite normal vision, intelligence, and other cognitive skills. Individuals with CP generally have great difficulties recognizing faces of other people, including their friends and family members, and can even have problems recognizing their own face. CP is a puzzling disorder as these individuals do not have frank neurological damage and, yet, they do not attain mastery of face recognition incidentally over the course of development (for a review, see Behrmann and Avidan, 2005). Of importance to vision science, CP offers a unique window into understanding the psychological and neural mechanism of face processing and, as such, this neurodevelopmental condition has received considerable attention recently.

Unlike acquired prosopagnosia (AP) which results from explicit brain damage and is rare, CP is more common in the population at large (approximately 2% of prevalence rate) in both the Caucasian (Kennerknecht et al., 2006), and non-Caucasian population (Kennerknecht et al., 2007, 2008), and runs in some families (de Haan, 1999; Grüeter et al., 2007; Johnen et al., 2014). Much of the recent research has explored the neural basis of CP and has identified differences, relative to controls, in the distributed face network. These differences are apparent to a greater degree in the more extended/anterior portions of the network than in the more core/posterior regions (Avidan and Behrmann, 2014; Avidan et al., 2014; but see Furl et al., 2010 for a different finding). Studies that explore the psychological or computational basis of CP have largely focused on the failure of these individuals to process visual information holistically and the goal of this study is to explore this further.

## Holistic processing (HP) of faces

Given that all faces differ only slightly in the shape and size of facial features which are arranged in the same top-heavy configurations, the spatial relations among these features is considered particularly important for face recognition. In line with this idea, it has been suggested that facial features and their spatial relations are processed holistically (for a review, see Maurer et al., 2002; Cheung and Gauthier, 2010); in other words, there is obligatory or non-independent encoding of all parts of the face and the parts cannot be ignored (for review of recent evidence and perspectives, see Richler and Gauthier, 2014). Moreover, not every observer engages holistic face processing to the same degree and individual differences in HP may have a significant impact on face recognition and may even be used to predict face recognition skills (Richler et al., 2011; Wang et al., 2012).

Many measures of HP have been developed and converging evidence from the face inversion task (Yin, 1969), the part-whole task (Tanaka and Farah, 1993) and the composite face task (Young et al., 1987; Richler et al., 2008) all support the idea that, in normal object perception, faces are processed in a more holistic fashion than other types of objects. Also, expertise with a class of objects can confer the need for HP of homogeneous exemplars (Richler et al., 2009) and HP emerges over the course of development (Scherf et al., 2009). Although there is still considerable debate in the literature concerning the relationship between HP and configural processing, we adopt an operational definition such as that articulated by Richler and Gauthier (2014; see also Amishav and Kimchi, 2010, for definitions) and examine the extent to which parts of a face are encoded in a mandatory and non-independent fashion.

In contrast with the reliance on HP evinced by normal observers, impaired HP and an over-reliance on featural processing are frequently reported in individuals with CP (e.g., Levine and Calvanio, 1989; Avidan et al., 2011). For example, CPs do not show the expected decrement in performance in inverted vs. upright faces (Behrmann et al., 2005). CPs also do not show normal performance in the context of the composite face paradigm. In the standard horizontal composite design, participants make same/different judgments of one half of two faces (say the top half) and the two halves of a face can be either misaligned or aligned. The signature of HP, known as the *composite face effect*, refers to the adverse impact on matching when the two relevant halves are the same (the top halves are identical across the two faces or the bottom halves are identical) and the two irrelevant halves are different, and this interference from the unattended half is greater when the face halves are aligned rather than when they are misaligned (see Rossion, 2013 for a review). That is, when the face halves are aligned, the interference from the irrelevant halves convincingly demonstrates that face processing is “holistic”: observers cannot help but process information about the unattended portion of the face, even if it is task-irrelevant. This interference is not apparent to the same extent in the misaligned trials indicating that the face is not perceived holistically when the parts are not in their usual configuration.

Interestingly, unlike the pattern described above, individuals with CP do not make false alarms in the horizontal composite task and do not show the increased interference from the aligned compared with the misaligned unattended half face (Avidan et al., 2011; Palermo et al., 2011). Instead, they perform more veridically than the controls (faster RTs and fewer false alarms), remaining immune to the contribution of the unattended aligned half, and thereby reflecting the deficit in HP in CP. Whether this holistic deficit is true for all CP individuals is unclear. For example, Le Grand et al. (2006) showed that, on a standard composite face task (attend to top or bottom half of face), 7 of their 8 CPs exhibit a composite face effect that is not differentiable from controls.

The failure of CP individuals to apprehend all parts simultaneously appears to extend beyond their ability to encode face parts holistically. For example, in one recent study (Tanzer et al., 2014), CP individuals were asked to judge the width of visually presented rectangles while ignoring their irrelevant height, or to judge changes in width while height remained constant in the context of a Garner speeded classification task. While controls exhibited the expected Garner interference, no such interference was observed for the CPs, indicating impaired HP of integral, non-facial shape dimensions. Both CPs and controls exhibited the same level of Garner interference when the task was changed to reporting non-shape dimensions (in this case, color). These findings indicate a deficit in holistic integral perception of shape dimensions in CP (but see recent paper by Busigny et al., 2014 for argument on face-specific impairment in holistic processing).

It is also the case that some studies show that deficits in CP extend beyond configural processing *per se* as these individuals are also impaired at integrating featural and configural information (Kimchi et al., 2012), and show local superiority and precedence in a hierarchical Navon letter task (Behrmann et al., 2005). Perhaps, unsurprisingly, these deficits adversely impact shape perception more generally rather than just affecting face perception, and this more general perceptual disorder results in difficulties in subordinate-level object discrimination, as well (Behrmann et al., 2005; Garrido et al., 2008). We note that, although there is a growing consensus that HP is affected in CP, this may not be true of all individual cases. As noted previously, most CP individuals in Le Grand et al. (2006) showed a normal composite effect. Also, DeGutis et al. (2012) reported that the inversion and scrambling of face images produced comparable deficits in CPs and controls, suggesting that both groups use holistic processing and configural information to recognize gender. Also, in some studies, CP participants exhibited the typical global superiority in the Navon compound letter task, assumed to tap into higher-order componential processing, as well (Duchaine et al., 2007b; but see Avidan et al., 2005 for impaired global perception in CP). The crux of the current study is to explore HP in CP further with use of a fine-tuned, novel paradigm, as described below, and to characterize this ability both at the group level and the level of each CP individual.

## Hemispheric lateralization of faces in CP

In addition to characterizing HP in CP, here, we examine an additional aspect of their behavior concerning possible differences in hemispheric specialization between CP and controls. There is a general consensus in the field that face perception and holistic processing are more strongly mediated by computations of the right hemisphere (RH) than the left hemisphere (LH). For example, Rossion et al. (2000) found that the RH was activated to a greater extent when participants matched whole faces than face parts whereas this pattern of activity was reversed in the LH homologous region (see also Meng et al., 2012, for differences in hemispheric computations in face perception). Whether CP individuals show a difference in hemispheric profile remains to be determined.

To date, there have been very few detailed explorations of differences in hemispheric specialization in CP vs. controls. Hasson et al. (2003) reported that their CP participant, YT, evinced activation in left lateral occipital (LO) cortex that was more than 1 SD outside the normal range, although they went on to show, using a laterality index, that this difference was unlikely to be associated with YT's face perception difficulty as some of the normal observers showed the same bias toward LH activation. Of course, the absence of a difference in the RH goes against the idea of a disadvantage in the preferential RH HP but nevertheless, the subtle LO difference in CP prompts us to explore this issue further. It is also the case that Avidan et al. (2014) noted a slight difference between CP and controls in hemispheric organization: specifically, they showed that there was greater activation (but not number of voxels or any other dependent measure) in CPs in the left superior temporal sulcus (STS) compared with the controls. Additionally, the right, but not the left occipital face area (OFA), was slightly larger in the controls than in the CP although the activation profiles were not dissimilar across the two groups. Together, these subtle atypicalities, although inconsistent across dependent measures, sides and studies, lead us to examine hemispheric differences in CP more closely in our characterization of HP.

## The current study

In the current study, we adopt a novel paradigm, the vertical composite task, and explore further the manner and extent to which individuals with CP are impaired at HP and whether this impairment is differentially modulated by hemispheric lateralization, relative to controls. The design of this novel task is a modification of the standard (horizontal) composite task, which has been used extensively to uncover HP of faces under a variety of contexts and manipulations (for overview, see Richler and Gauthier, 2013; Rossion, 2013), and specifically, it is designed to permit us to examine the hemispheric effects, as well.

**Figure 1:**
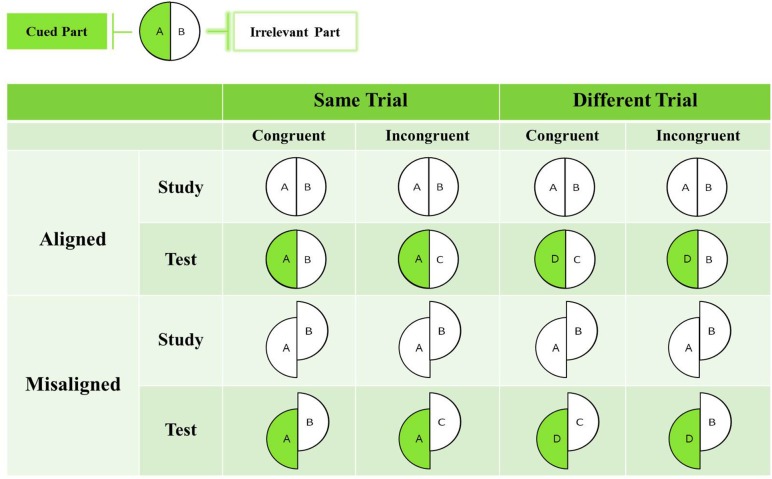
**Schematic diagram depicting the left-right composite paradigm.** In this example, the cued part is on the left (with a green/shaded background) and the irrelevant part is on the right (with a white background). The format of the study and test faces can be either both aligned or both misaligned. Participants are instructed to make a same/different judgment based on the cued part in the study face and the test face, and to ignore the other irrelevant part. In congruent trials, the study and test face halves can both be the same (AB→AB) or different (AB→DC). In incongruent trials, a change can occur either in the irrelevant part (AB→AC) or in the cued part (AB→DB).

To explore HP and its hemispheric effects in CP at both the group and the individual level, we examine whether the unattended half of the aligned/misaligned face influences performance on the attended half of faces that are halved vertically. Thus, we examine whether there is any effect on performance from the uncued half-face when participants are cued to the left (right visual field) or right (left visual field) of the chimeric face stimulus. To this end, we created left-right composite (or chimeric) faces by pairing the left half of one face with the right half of another face of the same gender and race. Figure 1 shows a schematic depiction of the paradigm.

This paradigm potentially affords us several advantages over the standard horizontal composite task. Bisecting the face along the horizontal vs. vertical dimension may make a difference to face perception. CK, an acquired agnosic individual who was able to recognize faces much better than objects, was able to identify famous faces much better when the faces were halved down the midline (and the two halves were misaligned) than when the bisection and misalignment was along the horizontal meridian (Moscovitch et al., 1997). Additionally, this paradigm permits us to examine the relative contribution of the left and right hemispheres to HP. This task is based on the rationale of the “chimeric face effect” (for example, see Indersmitten and Gur, 2003): when a chimeric face is presented over central fixation, observers show a robust preference to select chimeric faces made from two left sides of the original face as being more similar to the original face than chimeric faces made from two right sides (with the left side usually projected to the RH) (Gilbert and Bakan, 1973; Brady et al., 2005). This relative RH advantage for faces is so robust that it is even observed in non-human primates (Dahl et al., 2013) and the vertical composite task is motivated by this chimeric technique. Here, we combine the “chimeric face” technique in which two face halves are paired (along the vertical midline here) with the established composite face paradigm to explore the hemispheric basis of HP in the normal and CP observers.

We adopt the complete version of the composite task here (for a review, see Gauthier and Bukach, 2007, for recent exchange of opinions, see Richler and Gauthier, 2013; Rossion, 2013), which includes both congruent trials in which the relevant and irrelevant halves lead to the “same” response (i.e., both are same or both are different), and incongruent trials in which the relevant and irrelevant halves elicit a “different” response. In the example of Figure 1 in which the cued part is on the left (with a green/shaded background), the format of the study and test faces can be either both aligned or both misaligned. In addition, the study and test face halves can be either both the same/different (“congruent condition,” e.g., study face AB is followed by test faces AB or by test face DC), or one half is different between study and test (“incongruent condition,” e.g., study face AB is followed by test face AC). Although we expect performance differences between congruent and incongruent conditions (i.e., the “congruency effect”—akin to a Stroop-type of interference), the critical result, generally taken as an indicator of HP, is the interaction between alignment and congruency. That is, HP is defined as aligned (congruent–incongruent) d′–misaligned (congruent–incongruent) d′. Based on our predictions, we expect to observe a difference in the magnitude of HP (i.e., interaction between alignment and congruency) in controls and in CPs. Using this exact paradigm, we have previously obtained evidence for a composite effect in control participants (Liu et al., in press) and, as such, have verified the efficacy of the vertical composite task for uncovering HP. We note that in the controls, there was no modulation of the HP by hemisphere as we might have predicted given the evidence for greater RH involvement in HP. We have suggested several reasons why this interaction with hemisphere might be absent, but the pertinent question here is whether the CP individuals differ in their hemispheric contribution to HP relative to the controls.

## Methods

### Participants

All participants reported normal or corrected-to-normal vision and all were right-handed according to their responses to the Edinburgh Handedness Inventory (Oldfield, 1971). Informed consent was obtained prior to the start of the experiment and the protocol was approved by the Institutional Review Board at CMU.

#### Congenital prosopagnosics

Eight individuals with CP (age range 18–57 years, mean age = 36.6) participated in the study. Seven were tested in Pittsburgh, PA and one was tested in Nashville, TN. All CPs reported substantial life-long difficulties with face recognition and this impairment was confirmed by poor performance in both the Cambridge Face Memory Test (CFMT) and a famous face questionnaire used successfully to differentiate CP from controls in previous studies (Avidan et al., 2011) (see Table 1).

**Table 1:**
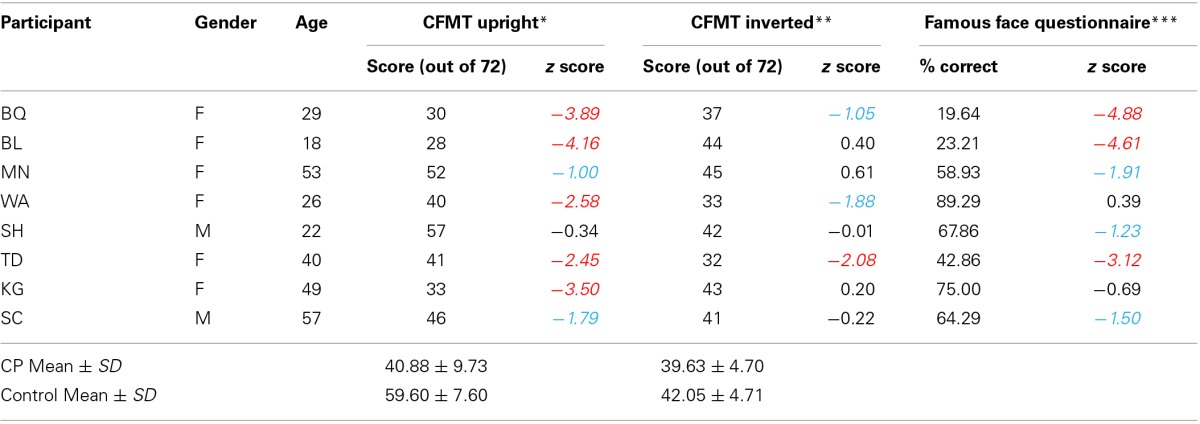
**Biographic details and results (raw values and *z* scores) of face perception measures for 8 CP individuals**.

Color code: z-scores that exceed 2 SDs are denoted in red italics and z-scores that are between 1 and 2 SDs are denoted in blue italics.

^*^Calculation of CFMT Upright z-score is based on the 20 control data provided in Duchaine et al. (2007a,b), M = 59.6 (out of 72 responses), SD = 7.6.

^**^Calculation of CFMT Inverted z-score is based on the 20 control data provided in Duchaine and Nakayama (2006), M = 42.05 (out of72 responses), SD = 4.71.

^***^Calculation of Famous face questionnaire z-score is based on the control data reported in Avidan et al. (2011), M = 84.1 (%correct), SD = 13.2.

#### Control participants

Two groups of control participants were recruited from the Pittsburgh community. One group consisted of thirty-two Caucasian students (mean age = 22.3 years, 12 M and 20 F) from Carnegie Mellon University (CMU) who participated in the study for course credit. The data from these individuals are reported in our previous study (Liu et al., in press). The other group consisted of eight individuals, age- and gender-matched to the CPs (age ± 3 years), recruited from the Pittsburgh community. As evident below, there are no differences in the performance of these two control groups and so we aggregate the data to obtain a large sample against which to benchmark the CP data.

### Materials and apparatus

**Figure 2:**
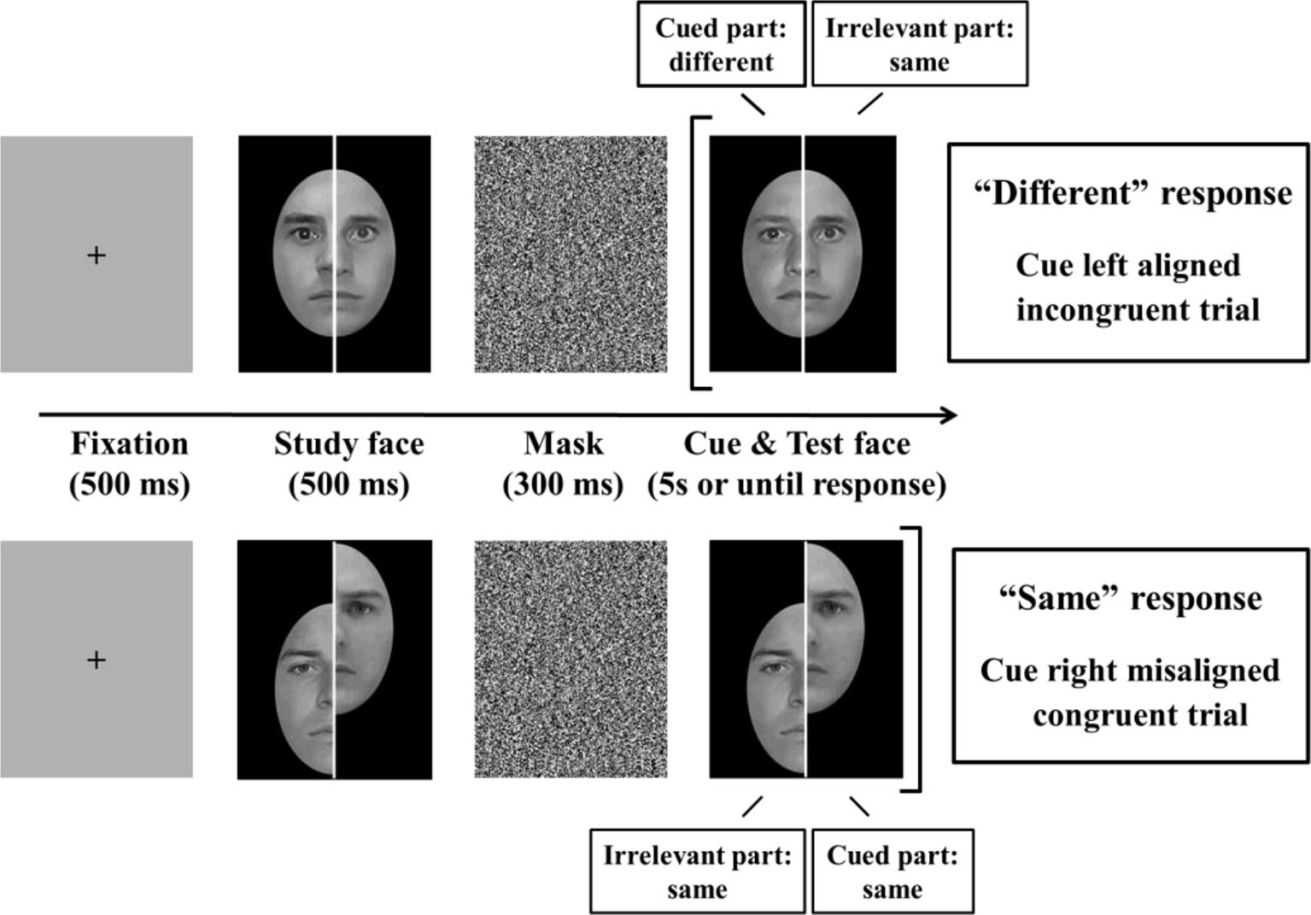
**Two sample trials of the vertical composite task.** As depicted here, a trial proceeds from fixation (left of image) to response (right of image). Participants were shown a composite face (study), which was masked, and were required to indicate whether the cued^2^ part in the subsequent test face was the same or different as the same half (left or right) in the study face. **Top:** a cue-left aligned incongruent trial. **Bottom:** a cue-right misaligned congruent trial.

The composite stimuli were created from 40 front-view Caucasian male faces (stimuli from Tanaka Lab) with neutral expressions and without hair or glasses. All faces were converted to grayscale images. Each face was approximately 170 pixels in width and 240 pixels in height and was fitted onto a uniform 320 × 420 pixel black background. To ensure that the task could not be performed based purely on facial symmetry (e.g., one eye is higher than the other, larger proportion of mouth on the right side), within each race, the twenty faces were subdivided into five groups of four similar faces based on prior ratings^1^. Each composite face was then created by pairing the left half of one face with the right half of another face from the same group. A 3-pixel-thick vertical white line was inserted at the center of the face to form a gap between the left- and right-half face. See Figure 2 for examples of a cue-left aligned incongruent trial and a cue-right misaligned congruent trial. Within each group, the positions of the eight face halves (left and right halves of the four faces) were rotated through a partial Latin square design such that one composite face was never studied again throughout the experiment. Two misaligned versions were included to counterbalance the up/down position of the left and right sides of the composite face: each misaligned composite face was created by moving the left half up or down approximately 80 pixels (around one third of the face).

For CP and their age- and gender-matched controls, stimuli were displayed on a 14″ laptop with a resolution of 1366 × 768 pixels and 60-Hz frame rate. These two groups of participants viewed the display from a distance of approximately 40 cm (although this was not fixed), and the face on the screen was 4 cm wide and 5.5 cm high; thus, each face subtended about 5.5° horizontally and 7.9° vertically. For the student control group, stimuli were displayed on a 20″ monitor with a resolution of 1680 × 1050 pixels and 60-Hz frame rate. Participants viewed the display from a distance of approximately 50 cm, and the face on the screen was 4.4 cm wide and 6.2 cm high; thus, each face subtended about 5° horizontally and 7° vertically.

### Design

This study had one between-subject variable: participant group (CP vs. control; see below for details on combining two control groups), and three within-subject variables: alignment (aligned vs. misaligned), congruency (congruent, incongruent), and visual field (left vs. right of test face). The dependent variable was recognition performance (d′).

### Procedure

The sequence of displays in a single trial is illustrated in Figure 2. Each trial began with a black fixation cross presented at the center of the gray screen for 500 ms. After that, a study composite face was shown for 500 ms, followed by a 300-ms mask. A test composite face, together with a square bracket cueing which half of the face (left or right half) was to be judged, was then displayed for 5 s or until a response was made (whichever came first). Participants were asked to judge whether the cued half in the test composite was identical or not to that in the study composite. Participants were instructed to respond as quickly and as accurately as possible by pressing “F” and “J” on the keyboard. The mapping of the key response was counterbalanced across participants. The aligned and misaligned trials were blocked and the experiment consisted of eight blocks of 80 trials each, resulting in a total of 640 trials. The experiment took around 35 min to complete (although some CP individuals took quite a bit longer to complete this). Each participant completed a practice session of 24 trials (consisting of both aligned and misaligned conditions) prior to the experiment. Practice data were checked and, in very rare cases, when accuracy fell below 60% correct, the participant was asked to complete one more practice session before proceeding to the experiment.

## Results and discussion

Preliminary analyses comparing the discrimination performance (d′) between the 8 matched controls and the 32 college student controls revealed no main effect of group (*p* > 0.05), or interaction of any other factor with group (*p* > 0.05). Therefore, we judged our matched control group to be a representative sample of observers with normal face perception and merged their data with those of the larger control group so as to have a widely-sampled distribution of normal performance against which to compare the CPs.

### CP vs. control

A four-way mixed ANOVA on discrimination performance (d′), with alignment (aligned, misaligned), congruency (congruent, incongruent), and visual field (cueing left, right of the test face) as within-subjects factors, and participant group (CP, control) as the between-subjects factor revealed a significant effect of group [*F*_(1, 46)_ = 41.639, *p* < 0.001]. As expected, the CP individuals exhibited poorer discrimination performance relative to controls (controls: mean *d*′ = 1.33, *SD* = 0.53, CP: mean *d*′ = 0.57, *SD* = 0.37), confirming their status as impaired at face perception. There was also a significant interaction of congruency × group, *F*_(1, 46)_ = 9.931, *p* = 0.003, but not with any other factors alone [visual field × group: *F*_(1, 46)_ = 0.657, *p* = 0.422, alignment × group, *F*_(1, 46)_ = 3.546, *p* = 0.066], or with the combination of any two or three factors [alignment × congruency × group, *F*_(1, 46)_ = 2.758, *p* = 0.104, visual field × alignment × congruency × group, *F*_(1, 46)_ = 2.256, *p* = 0.140]. We examined the basis of the congruency by group interaction by carrying out a paired-samples *t*-test, comparing performance in congruent vs. incongruent trials separately for the CP and control groups. A significant congruency effect was observed in both the control group, *t*_(39)_ = 11.851, *p* < 0.001, and in the CP group, *t*_(7)_ = 3.155, *p* = 0.016, somewhat attenuated in the latter case perhaps because of reduced statistical power relative to controls. According to previous research (Bukach et al., 2006; Richler et al., 2008; Curby et al., 2013; but see Rossion, 2013 for a counterargument), the congruency effect alone can be indicative of evidence for HP, and therefore, the observed congruency by group interaction confirms a difference between the CP and control observers.

**Figure 3:**
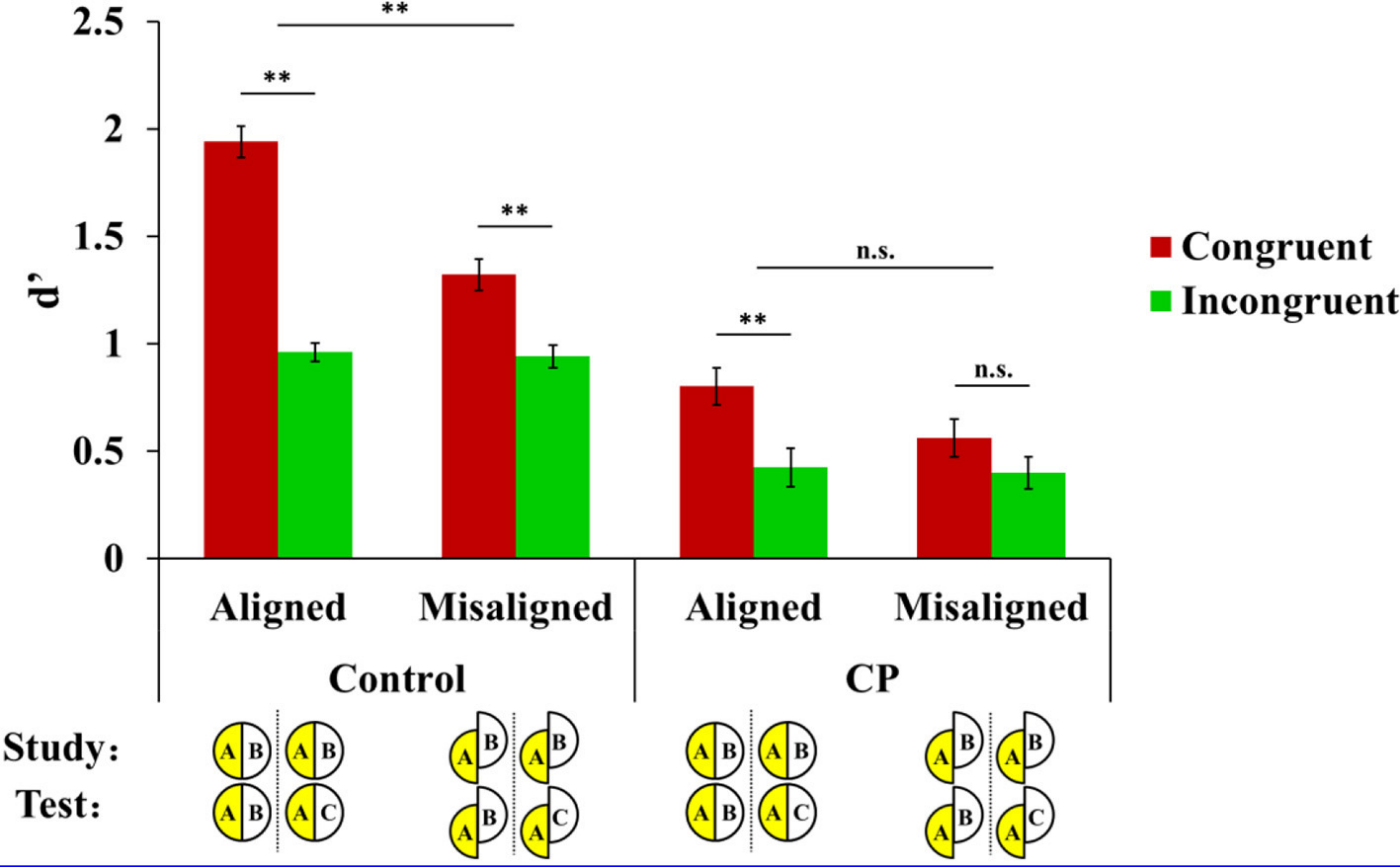
**Mean performance (*d*′) for the control group (left) and CP participants (right) on congruent and incongruent trials for aligned vs. misaligned faces.** Note that although the schematic display shows the example of cueing the face half in the left visual field, the data were collapsed across visual fields. Error bars show ±1 standard error of the mean (^**^p < 0.01).

Note also that because of our a priori hypotheses and the fact that some of the higher-order interactions are trending toward statistical significance, we undertook further investigation within each group so as to elucidate any possible differences in response profile per group. As laid out in the rationale, the alignment by congruency interaction is the most stringent criteria for HP. Because of this a priori prediction and the possibility that unbalanced sample size might have concealed the potential HP by group interaction, we investigated the alignment by congruency interaction separately in CP and in controls. To this end, we conducted a 2 × 2 (alignment × congruency) repeated-measures ANOVA on discrimination performance (d′) separately within the CP group and within the control group. We also excluded the factor of visual field from further analysis because it failed to show a main effect or interaction with any factors in the previous ANOVA, suggesting equal participation of both hemispheres in the left-right composite face task across all groups. Performance (d′) on congruent and incongruent trials in the aligned and misaligned conditions is plotted separately for controls and CP in Figure 3.

#### Controls

In the control data (aggregated over 32 college student controls and 8 matched controls), there was a significant alignment by congruency interaction [*F*_(1, 39)_ = 41.488, *p* < 0.001], indicative of HP of left-right composite faces. In other words, judgment of the cued half is strongly influenced by the irrelevant half when faces are aligned, and this influence is reduced when faces are misaligned. In addition, the main effect of congruency was significant, *F*_(1, 39)_ = 150.191, *p* < 0.001. The main effect of alignment was also significant, *F*_(1, 39)_ = 52.603, *p* < 0.001, with better performance in the aligned than misaligned condition. A follow-up paired samples *t*-test revealed that the enhanced performance in the aligned vs. misaligned condition was only observed in congruent trials, *t*_(39)_ = 10.091, *p* < 0.001, where relevant and irrelevant halves led to the same response (i.e., both are same or both are different). This means that the response on the relevant half is facilitated by the irrelevant half because their responses are congruent, and this facilitation is larger in the aligned than misaligned condition. In contrast, there was no difference between performance in the aligned than misaligned condition in incongruent trials, *t*_(39)_ = 0.333, *p* = 0.741, where relevant and irrelevant halves elicit different responses (same vs. different response).

#### CP

In contrast with the profile of the control participants, the composite face effect was absent in the CP data, evidenced by a non-significant interaction between alignment and congruency, *F*_(1, 7)_ = 2.095, *p* = 0.191. Note that the alignment by congruency interaction was significant in the eight age- and gender-matched controls, *F*_(1, 7)_ = 5.723, *p* < 0.05, and therefore, the absence of this interaction in the CP group was not due to a lack of statistical power. Note that because in the upright version of CFMT in Table 1, MN (*z*-score = −1.00), SH (*z*-score = −0.34) and SC (*z*-score = −1.79) performed within 2SD of the normal range, here we further used a leave-one-out procedure (MN/SH/SC) and repeated the analysis of the composite face effect. The pattern of alignment by congruency interaction was not affected by this procedure [without MN: *F*_(1, 6)_ = 1.428, *p* = 0.277; without SH: *F*_(1, 6)_ = 2.907, *p* = 0.139; without SC: *F*_(1,6)_ = 4.391, *p* = 0.081] and therefore we decided to include all these three CPs in the final analysis. In addition, CPs showed a significant main effect of alignment, *F*_(1, 7)_ = 6.879, *p* = 0.034, with better performance in the aligned condition than in the misaligned condition and a main effect of congruency, *F*_(1, 7)_ = 9.981, *p* = 0.016, with higher discrimination sensitivity for congruent trials than incongruent trials. Nevertheless, the magnitude of both effects of alignment and congruency was much smaller than that of the control group. See Figure 3 for a comparison among CP and the two control groups.

Because of the possible heterogeneity in HP in CP individuals, we also undertook an analysis of performance at the individual level and we report these data below. To do so, Crawford's modified *t*-test (Crawford and Howell, 1998; Crawford and Garthwaite, 2002) was used to assess the performance difference between each CP's score and the control sample. To this end, we created two indices critical for this task: specifically, for each participant, the congruency index was created by subtracting performance in the incongruent trials from that in the congruent trials, i.e., congruency index = congruent *d*′–incongruent *d*′, and the holistic processing index was created by subtracting the difference between congruent and incongruent trials in the misaligned condition from that in the aligned condition, i.e., (aligned congruent *d*′–aligned incongruent *d*′)–(misaligned congruent *d*′–misaligned incongruent *d*′).

**Figure 4:**
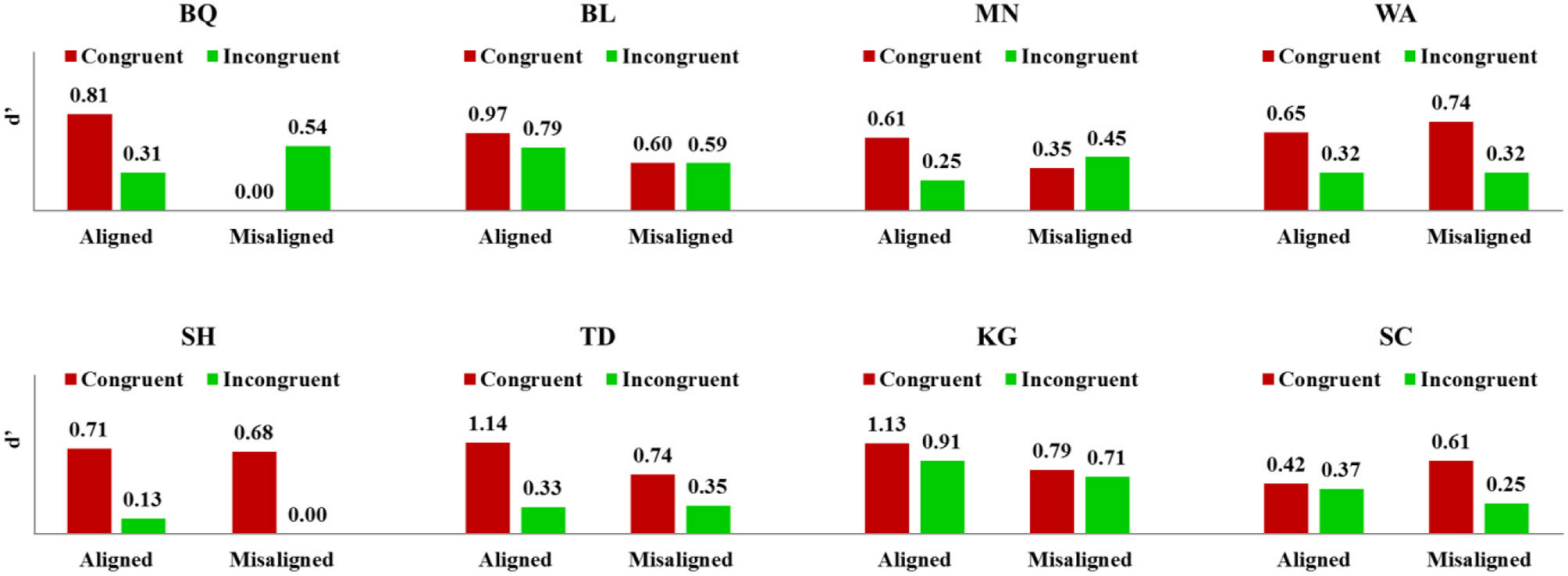
**Performance (*d*′) for each individual CP participant on congruent and incongruent trials for aligned vs. misaligned faces where the cued half was collapsed across visual fields (note that the y-axis varies across participants)**.

As can be seen from Table 2, five out of eight CPs showed significant impairment in the holistic processing index from the Crawford's *t*-test (*p* < 0.05, two-tailed) and the individual data from each CP participant is shown in Figure 4. We note that three CPs (BQ, MN, and TD) do not show a statistically significant HP effect and two of these three, MN and TD, show a trend in the right direction and it is only participant BQ who shows a different profile. Closer scrutiny of BQ's data shows higher *d*′ for the aligned congruent than aligned incongruent trials, but his *d*′ for misaligned congruent trials is 0.00, which is very unusual compared to the other CPs and because of this, there is no significant composite effect. Based on these results, we can conclude that 7 CP individuals (to a greater or lesser degree) show a reduction in HP of faces.

**Table 2:**
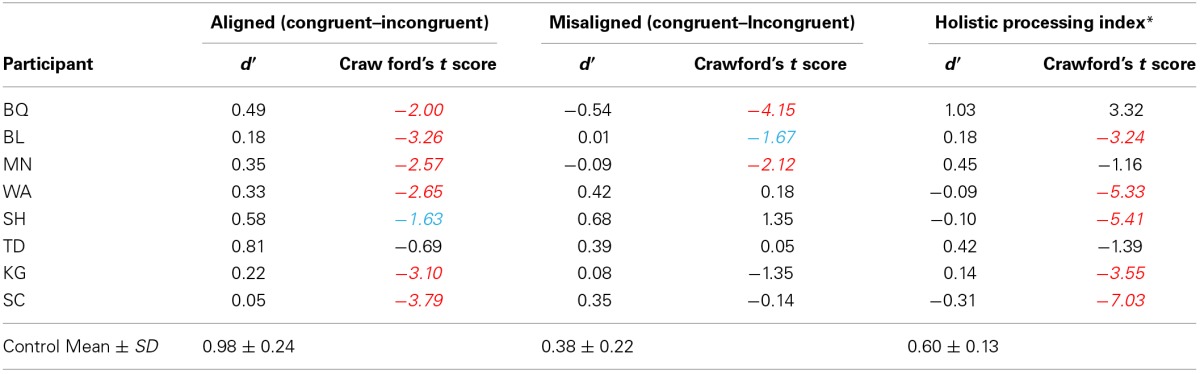
**Crawford's *t*-test scores of the aligned congruency index, misaligned congruency, and the holistic processing index for each individual CP participant**.

Color code: Crawford's t-scores (negative only) with p values < 0.05 (two-tailed) are denoted in red italics and those with p values < 0.05 (one-tailed) are denoted in blue italics.

^*^Holistic processing index is calculated as aligned (congruent–incongruent) d′–misaligned (congruent–incongruent) d′.

For comparison purposes, we also computed the HP scores for each of the 40 controls using a leave-one-out procedure (compute means based on all controls with the exception of the target control and then assess the status of the left-out control relative to the mean and distribution of the group and this was repeated for each participant). Of the controls, 13 out of 40 do not show a Crawford significant HP result relative to the control group mean. In fact, there has been some recent consideration of the variability of performance (and lack of consistency at an individual level) of the standard composite effect (Ross et al., in press) and some discussion on ways to enhance the reliability and robustness of the finding, which holds strongly at the group level.

## General discussion

Congenital prosopagnosia is an intriguing neurodevelopmental disorder in which individuals are impaired at face perception apparently from birth, in the absence of any sensory or intellectual deficits. The reigning hypothesis is that the psychological mechanism that underlies the difficulty in face processing in these individuals is one in which holistic processing (HP) is impaired. Much research has provided evidence in support of this hypothesis including data showing that CP individuals do not show the expected inversion effect (Rouw and de Gelder, 2002; Behrmann et al., 2005; Avidan et al., 2011), do not show a global superiority effect in a Navon-compound letter task (Behrmann et al., 2005; Avidan et al., 2011) and do not show HP in a standard composite top-down task (for example, Ramon et al., 2010; Avidan et al., 2011; Palermo et al., 2011). Closer scrutiny, however, reveals several counterexamples. For example, Le Grand et al. (2006) reported that, of the eight CPs who participated in their study, surprisingly, only one CP showed an abnormal composite effect. Additionally, Susilo et al. (2011) reported that the CP in their study showed a composite effect across three different tasks (naming and two same/different judgments). Also, Schmalzl et al. (2008) tested a family of seven developmental prosopagnosia (DP) individuals (spanning four generations) and reported that only four individuals failed to show the normal composite effect, and, finally, Williams et al. (2007) found a normal composite effect in a case of DP. In light of the contrasting results reported to date, the purpose of the present investigation was to examine further the nature of holistic processing in CP vs. matched controls using a new left-right composite face task. In addition, we wished to assess possible differences between the groups in hemispheric modulation of the composite effect and to document the magnitude of the HP effect at an individual level. The vertical composite task was modeled after the well-known face chimeric effect in which two half faces presented to the left and right of fixation reveal superior processing of the half face that occupies the left visual field (right hemisphere).

At a group level, unlike the control individuals (*n* = 40, comprised of matched controls and a large group of non-matched controls), the CP individuals did not show an interaction of congruency × alignment. Moreover, the CP performance (in *d*′) is significantly lower than that of the controls, some of whom are directly pairwise matched with the CP individuals. Of interest, the CP group does show significantly poorer performance when the faces are misaligned compared with when they are aligned, reflecting residual sensitivity to first-order properties of the face (Maurer et al., 2002). The CPs also show a main effect of congruency, with higher discrimination sensitivity for congruent trials than incongruent trials. Although some accept this signature as a measure of HP, in that the unattended face half influences performance on the attended face half, the congruency effect is not considered the golden metric of HP (the alignment × congruency interaction).

Given the heterogeneity of individual CP cases, as reviewed above, we assessed each participant individually. The majority of CP individuals performed outside the normal range when a case-by-case analysis was done (7 out of 8 participants), further confirming the difficulties in HP. However, we note that almost a third of the controls also failed to show a composite effect when the individual control data were assessed (see Ross et al., in press for more detailed discussion of the reliability of the composite face task).

Surprisingly, but interestingly, we observed no differences between the controls and the CP in terms of modulation of the composite effect by hemisphere, i.e., performance was the same independent of whether the cued face half fell in the right or left visual field. While we were surprised by the absence of hemispheric modulation in the controls (see Liu et al., in press) given how closely this paradigm mirrors the known chimeric face result, of interest here is that the CPs, too, show no hemispheric modulation.

In sum, the CP individuals performed more poorly than the controls in a task of face matching that taps HP. These results support the claim that a breakdown in holistic processing may be at the basis of CP. The paradigm we designed appears to be effective in uncovering this difficulty and confirms the deficit in HP as noted on many previous reports (for example, Avidan et al., 2005; Ramon et al., 2010; Palermo et al., 2011; Kimchi et al., 2012). We note that a decrement in HP in CP may be quite ubiquitous and may even be evident in the failure of these individuals to determine aspect ratio (conjoint representation of the length and the width of rectangles) (Tanzer et al., 2014) in the ability to configurally represent other non-face stimuli too (Lange et al., 2009).

It is also the case that CPs may not only be impaired at HP but may even show some deficits in featural processing as well. For example, in the context of a Garner speeded-classification task using facial stimuli, unlike in the controls, the CP group exhibited no Garner interference in either the featural or the configural judgments. When classifying upright faces that varied in features (shape of eyes, nose, and mouth) and configuration (intereyes and nose–mouth spacing), the CPs could attend to configural information and make configural judgments without interference from irrelevant variation in featural information; similarly, they could attend to featural information and make featural judgments without interference from irrelevant variation in configural information. This pattern of performance, which is in clear contrast to the symmetric Garner interference observed in matched controls (and in young controls), indicates that featural information and configural information are separable in CP's upright face processing. That is, CPs do not perceive and process faces holistically. Rather, CPs process facial features and facial configuration independently.

Taken together, the findings of the current study are consistent with previous reports of altered visual perception in CP, specifically in the domain of HP. With this basic understanding of the possible underpinnings of the impairment, there have been some recent attempts to remediate the face processing deficits in CP with specific focus on retraining HP. DeGutis et al. (2007) devised a behavioral task that required discrimination of faces by their spatial configuration. This task was completed repeatedly by a single prosopagnosic individual and interestingly, after extensive training, not only did the individual improve in behavioral performance but also evinced a face-selective N170 after training that was not evident pre-training. There was also an increase in functional connectivity between ventral occipital temporal face-selective regions (right occipital face area and right fusiform face area) post-training, as well. More recently, DeGutis et al. (2014) explored whether it is possible to enhance face processing in a large group of CPs using a 3-week online face-training program targeting holistic face processing. The trained CPs showed moderate but significant overall training-related improvements on measures of front-view face discrimination and some showed significantly increased holistic face processing to the point of being similar to that of unimpaired control subjects. The findings also showed modest but consistent self-reported diary improvements. Clearly, further work along similar lines will continue to add to our understanding of the underlying deficit in CP and ways in which this can be offset through intervention.

## Conclusions

Consistent with the suggestion that impaired HP may underlie CP's difficulty in face processing, using a novel left-right composite face paradigm, we observed normal HP in control observers but reduced HP in CP. In addition to the group level performance, detailed examination of individual level performance showed that most CP individuals evinced no HP although this was also true in the individual profiles of about one third of the controls. Contrary to our prediction on differential hemispheric contribution to HP, neither CP nor control group showed any difference in performance as a function of hemifield of the cued face half, suggesting equal participation of both hemispheres to HP. In conclusion, the present study verified the use of a novel left-right composite face paradigm, which may potentially contribute to the study of HP in individuals with normal face perception and atypical face perception.

### Conflict of interest statement

The authors declare that the research was conducted in the absence of any commercial or financial relationships that could be construed as a potential conflict of interest.

## Footnotes

^1^ A naïve observer at CMU grouped the Caucasian male faces in the database in a way that maximizes face similarity within each group. Each group contained up four faces and each face was used only once.

^2^ The black square brackets in the figure are for illustration purposes only. In the experiments, the cue was a yellow frame overlaid on top of the black outline of either the left or the right face half. We opted for the black square brackets here because the yellow frame would not stand out from the black background in the monochrome version of the figure.
